# Interferon-β Intensifies Interleukin-23-Driven Pathogenicity of T Helper Cells in Neuroinflammatory Disease

**DOI:** 10.3390/cells10082139

**Published:** 2021-08-20

**Authors:** Agnieshka Agasing, James L. Quinn, Gaurav Kumar, Robert C. Axtell

**Affiliations:** Department of Arthritis and Clinical Immunology, Oklahoma Medical Research Foundation, Oklahoma City, OK 73104, USA; a.m.agasing@gmail.com (A.A.); jmsqnn89@gmail.com (J.L.Q.); gaurav-kumar@omrf.org (G.K.)

**Keywords:** experimental autoimmune encephalomyelitis, T helper 17, interferon-beta

## Abstract

Interferon (IFN)-β is a popular therapy for multiple sclerosis (MS). However, 25–40% of patients are nonresponsive to this therapy, and it worsens neuromyelitis optica (NMO), another neuroinflammatory disease. We previously identified, in both NMO patients and in mice, that IFN-β treatment had inflammatory effects in T Helper (TH) 17-induced disease through the production of the inflammatory cytokine IL-6. However, other studies have shown that IFN-β inhibits the differentiation and function of TH17 cells. In this manuscript, we identified that IFN-β had differential effects on discrete stages of TH17 development. During early TH17 development, IFN-β inhibits IL-17 production. Conversely, during late TH17 differentiation, IFN-β synergizes with IL-23 to promote a pathogenic T cell that has both TH1 and TH17 characteristics and expresses elevated levels of the potent inflammatory cytokines IL-6 and GM-CSF and the transcription factor BLIMP. Together, these findings help resolve a paradox surrounding IFN-β and TH17-induced disease and illuminate the pathways responsible for the pathophysiology of NMO and MS patients who are IFN-β nonresponders.

## 1. Introduction

IFN-β remains a popular treatment for relapsing-remitting multiple sclerosis (RRMS). However, despite the clinical efficacy of IFN-β, approximately 25–40% of RRMS patients respond poorly to this treatment [1], and it consistently worsens disease in patients with neuromyelitis optica (NMO), a neuroinflammatory disease often misdiagnosed as MS [2,3]. These observations demonstrate that there are limitations to the therapeutic benefits of IFN-β.

IFN-β is a pleiotropic cytokine that has many effects on the immune system. Although the mechanism of action of IFN-β in MS is currently unclear, some have speculated that the efficacy of this therapy is achieved by inhibiting the function of T Helper (TH) 17 cells [4,5,6]. However, biomarker studies of MS and NMO patients, as well as animal experiments, have confounded this theory.

In the MS population, there are clear responders and nonresponders to IFN-β therapy. The differences in response in patients have been associated with therapeutic noncompliance [7], the development of neutralizing antibodies [8], as well as differences in the immunological signatures of patients [9]. We previously identified that the balance of TH1 to TH17 signatures determined the efficacy of IFN-β therapy. High TH1 signatures and high levels of IL-7 before initiation of therapy are associated with a good response to IFN-β [10,11]. Alternatively, elevated TH17 cytokine signatures in a subset of MS patients are associated with nonresponders [9,11,12]. In NMO, a disease with a strong association with TH17 signatures [13], patients have an increased frequency of relapses when treated with IFN-β [2,3,14].

We recently reported, in both NMO patients and in mice, that IFN-β treatment had inflammatory effects in T Helper (TH) 17 induced through the action of the inflammatory cytokine IL-6 [15]. However, other studies have shown that IFN-β inhibits TH17 [4,5,6]. This manuscript now provides data to reconcile the conflicting reports of IFN-β on the differentiation and pathogenic function of TH17 cells in neuro-autoimmune diseases.

## 2. Materials and Methods

### 2.1. Mice

Eight- to ten-week-old female C57BL/6J mice were purchased from Jackson Laboratory, housed in the Oklahoma Medical Research Foundation animal facility and treated in compliance with the Institutional Animal Care and Use Committee (IACUC).

### 2.2. Culturing Encephalitogenic T Cells

Donor C57BL/6 mice were subcutaneously immunized with 150 μg MOG_35–55_ peptide (Genemed Synthesis Inc., San Antonio, TX, USA) emulsified in Complete Freund’s adjuvant (5 mg/mL heat-killed *M. tuberculosis*). This was followed by an intraperitoneal (I.P.) injection of 250 ng of *Bordetella pertussis* toxin (List Biological Laboratories Inc., Campbell, CA, USA) in PBS at day 0 and day 2 post-immunization. Donor mice were sacrificed day 10 post-immunization, and spleens and lymph nodes were harvested and mechanically disrupted to obtain a single-cell suspension. 2.5 × 10^6^ cells/mL were stimulated for three days with MOG_35–55_ (10 µg/mL), IL-23 (10 ng/mL; R & D Systems, Minneapolis, MN, USA) and anti-IFN-γ (10 µg/mL; eBioscience, San Diego, CA, USA) in the presence or absence of IFN-β (100 U/mL; PBL, Novato, CA, USA) in complete RPMI 1640 (Gibco, Waltham, MA, USA). To block IL-6 signaling, cells were cultured with anti-IL-6 (10 μg/mL; BD Biosciences, Franklin Lakes, NJ, USA).

### 2.3. Adoptive Transfer EAE

C57BL/6J recipient mice were I.P. injected with 5 × 10^6^ cells and monitored daily for clinical scores. Paralysis was assessed using the following standard clinical score: (0) healthy, (1) loss of tail tone, (2) partial hind-limb paralysis, (3) complete hind-limb paralysis, (4) forelimb paralysis, and (5) moribund/dead. Transfer EAE mice were sacrificed at day 15, and spinal cords were fixed and sectioned for histological analysis using H & E and Luxol fast blue staining.

### 2.4. Isolation of CNS-Infiltrating Cells

Mice were perfused with PBS, and spinal cords were collected and homogenized through mechanical disruption. CNS homogenates were incubated with DNAse (5 µL/mL; Sigma) and collagenase D (4 mg/mL; Roche) at 37 °C for 1 h, and cells were isolated using a Percoll gradient.

### 2.5. Flow Cytometric Analysis of Cytokine Expression

Cells were stimulated with 50 ng/mL PMA (Sigma-Aldrich, St. Louis, MO, USA), 500 ng/mL ionomycin (Sigma-Aldrich), and monensin (BD Biosciences) for 4 h as described by the manufacturer’s protocol. Cells were then stained with CD4 (eBioscience) or CD19 (eBioscience), fixed and permeabilized with Cytofix/Cytoperm (BD Biosciences) and stained for IL-17 (BioLegend), IFN-γ (BioLegend) and/or IL-6 (eBioscience). Data was collected on LSRII (BD Biosciences) and analyzed using FlowJo software (Tree Star Inc., Ashland, OR, USA).

### 2.6. Analysis of Cytokine Production by ELISA

Culture supernatants were collected for ELISA. IL-17, IL-10, IL-6 and GM-CSF levels were assessed using anti-mouse ELISA kits (eBioscience).

### 2.7. RNA Isolation and Quantitative Real Time RT-PCR

RNA was extracted from cells with an RNAeasy Mini Kit (QIAGEN). cDNA was generated using an iScript cDNA synthesis kit (Bio-Rad, Hercules, CA, USA). qRT-PCR was performed using forward and reverse primers (see Table S1), cDNA and iQ SYBR Green Supermix (Bio-Rad) on a 7900HT Fast Real-Time PCR System (Applied Biosystems, Waltham, MA, USA). Sample reactions were carried out in triplicate. GAPDH was used as a reference gene, and the relative gene expression analysis was measured using the 2-ΔΔCt method [16].

### 2.8. Intracellular Staining of Phosphorylated STAT Proteins

Cells from the spleen and lymph nodes of MOG-immunized mice were cultured as described above. After 0, 15, 30 and 60 min, cells were fixed with 4% paraformaldehyde for 10 min, centrifuged at 1500 rpm for 5 min and permeabilized on ice with 100% cold methanol for another 10 min. Cells were then washed with PBS and stained with the following antibodies: anti-CD4 (eBioscience), pSTAT1 (BD Biosciences), pSTAT3 (BD Biosciences), pSTAT4 (eBioscience), pSTAT5 (eBioscience) and pSTAT6 (eBioscience) for flow cytometric analysis.

### 2.9. In Vitro TH17 Differentiation

Spleen cells from healthy mice were depleted of CD8^+^ T lymphocytes using CD8a (Ly-2) MicroBeads (Miltenyi Biotec) and cultured in two sequential phases. For TGF-Beta-dependent TH17 differentiation, cells were cultured (5 × 10^6^ cells/mL) with antibodies against CD3 (eBioscience; 10 µg/mL) and CD28 (eBioscience; 0.5 µg/mL) and the cytokines IL-6 (R & D Systems; 20 ng/mL) and TGF-β (R & D Systems; 1 ng/mL) in the presence or absence of IFN-β (100 U/mL) for three days. For IL-23-dependent TH17 differentiation, the cells initially cultured with IL-6 and TGF-β (without IFN-β) were washed and recultured for an additional three days with anti-CD3 (10 µg/mL), anti-CD28 (0.5 µg/mL), IL-23 (10 ng/mL) and anti-IFN-γ (10 µg/mL) with or without IFN-β (100 U/mL).

### 2.10. Statistical Analysis

Data are presented as means ± s.e.m., and statistical significance was determined using Mann–Whitney tests. In the case of three or more data sets, means were compared using the Kruskal–Wallis test with a Dunn’s multiple comparison correction. Differences were considered significant for *p* < 0.05. Statistical analyses were performed using Prism 6 (GraphPad, San Diego, CA, USA).

## 3. Results

### 3.1. IFN-β Potentiates the Pathogenicity of Myelin-Enriched TH17 Cells

**Figure 1:**
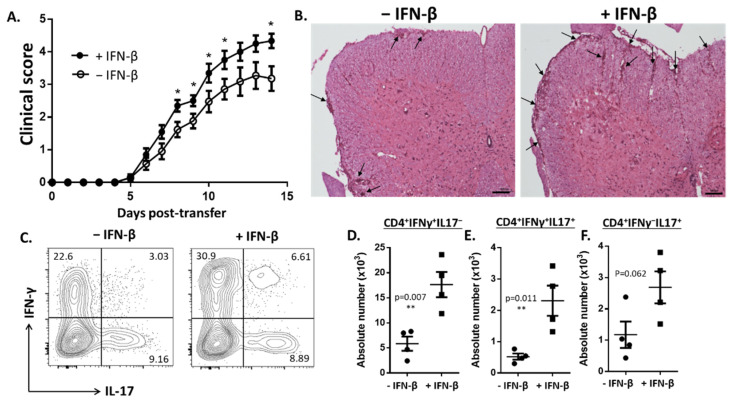
IFN-β increases the pathogenicity of IL-23-stimulated, myelin-enriched TH17 cells. (**A**) Clinical scores of mice with EAE induced by adoptive transfer of splenocytes cultured with MOG_35–55_, IL-23 and anti-IFN-γ in the presence (+) or absence (−) of recombinant mouse IFN-β (*n* = 20–21 mice per group from three independent experiments). (**B**) Histology of spinal cord sections from representative mice from the − IFN-β and + IFN-β groups. Sections were obtained 15 days post-transfer and stained with H & E. Arrows indicate demyelinating lesions in the brain and spinal cord. (**C**) Representative flow cytometry plots of CD4+ T cells expressing IFN-γ and IL-17 in the spinal cords of EAE mice, 15 days post-transfer. Absolute numbers of T helper cells that are (**D**) IFN-γ^+^IL-17^-^, (**E**) IFN-γ^+^IL-17^+^ and (**F**) IFN-γ^-^IL-17^+^ in the spinal cords of EAE mice. Error bars represent means ± s.e.m. Statistical analysis was performed using Mann−Whitney tests. *p* values < 0.05 were considered significant (** indicates *p* ≤ 0.01, * indicates *p* < 0.05).

Previous studies have shown that IFN-β treatment worsened TH17-induced EAE [11]. To understand how IFN-β increased TH17 disease severity, we assessed the direct effects that IFN-β stimulation had on the encephalitogenic capacity of IL-23-derived TH17 cells. We cultured donor cells with MOG_35–55_ and IL-23 in the presence or absence of IFN-β prior to the transfer into healthy recipient mice. Mice which received IL-23- and IFN-β-stimulated cells showed exacerbated disease scores compared to recipient mice that received cells stimulated with only IL-23 (Figure 1A). In agreement with the clinical scores, the histological analysis of spinal cords indicated that mice receiving IFN-β-stimulated cells had increased CNS-inflammation compared to mice receiving cells stimulated with only IL-23 (Figure 1B).

We next examined the production of IL-17 and IFN-γ in the CNS of recipient mice 15 days following adoptive transfer. In the spinal cords, mice receiving cells stimulated with IFN-β and IL-23 had significantly elevated numbers of IFN-γ^+^IL-17^-^ and IFN-γ^+^IL-17^+^ TH cells compared to mice that received cells stimulated with only IL-23 (Figure 1C–E). The differences in the numbers of IFN-γ^-^IL-17^+^ TH cells were nearly significant between both groups of mice (Figure 1F).

### 3.2. IFN-β Induces Inflammatory Cytokines in Myelin-Enriched TH17 Cells

**Figure 2:**
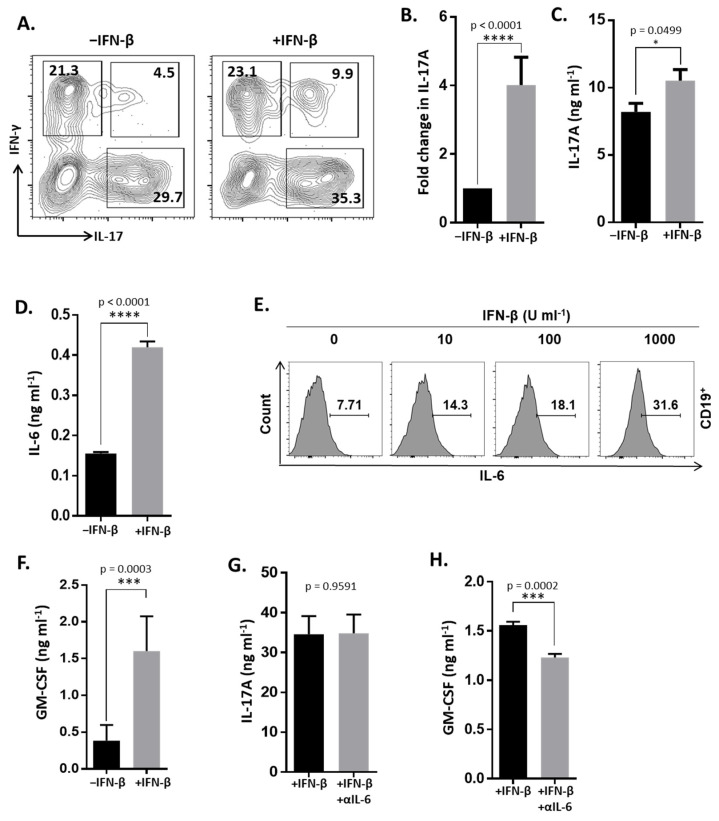
IFN-β induces inflammatory cytokine expression in IL-23-stimulated TH17 cultures. Spleen and lymph node cells of MOG-immunized C57BL/6J mice were cultured with MOG_35–55_, IL-23, and anti-IFN-γ in the presence or absence of IFN-β. (**A**) Representative FACS plots of CD4+ T cells expressing IFN-γ and IL-17. (**B**) Expression of IL-17 transcripts in the cultured cells measured by qRT-PCR. (**C**) Secreted IL-17 levels in the culture supernatants measured by ELISA. (**D**) Secreted levels of IL-6 in the culture supernatants measured by ELISA. (**E**) Intracellular IL-6 was measured in CD19^+^ B cells by flow cytometry. (**F**) GM-CSF in the culture supernatants measured by ELISA. The concentration of (**G**) IL-17 and (**H**) GM-CSF from culture supernatants from IFN-β ± α IL-6 stimulation were assessed by ELISA. Statistical analysis was determined using Mann−Whitney tests. *p* values < 0.05 were considered significant (**** indicates *p* < 0.0001, *** indicates *p* < 0.001, * indicates *p* < 0.05). Error bars indicate s.e.m.

It has been reported that IFN-β inhibits IL-17 expression [4,5,6]. However, we detected increased numbers of IL-17^+^ TH cells in the CNS of recipient mice that received IFN-β-treated cells. Therefore, we next determined the phenotype of the cells before they were transferred into recipient mice. Strikingly, we observed that CD4^+^ T cells treated with IFN-β and IL-23 had increased IL-17 expression measured by FACS, qRT-PCR and ELISA compared to CD4^+^ T cells treated with only IL-23 (Figure 2A–C). We also assessed the expression of other inflammatory cytokines. We observed that in IL-23-stimulated conditions, IFN-β significantly elevated IL-6, although the amounts were substantially lower than IL-17 (Figure 2D). T cells are not thought of as a major source of IL-6. In these culture conditions, we observed that IFN-β increased IL-6 production in B-cells in a dose-dependent manner (Figure 2E). Recently, it has been reported that GM-CSF is a key cytokine produced by TH cells which promotes neuroinflammation [17]. Here, we show that IFN-β significantly elevates GM-CSF expression in IL-23-stimulated conditions (Figure 2F).

IL-6 is a critical factor for TH17 differentiation and IL-17 expression [18]. Our data demonstrating that IFN-β increased the expression of IL-17 and IL-6 led us to hypothesize that IFN-β indirectly induced IL-17 through IL-6. We tested this hypothesis by inhibiting IL-6 in these cultures with a neutralizing antibody. We observed that blocking IL-6 expression had no effect on IL-17 expression (Figure 2G). However, we did find that IL-6 inhibition significantly reduced GM-CSF expression (Figure 2H). We recently reported that in vivo administration of IFN-β exacerbated paralysis and elevated GM-CSF^+^ TH cells in CNS mice with TH17-EAE [15]. Furthermore, the therapeutic blockade of IL-6 reversed these effects of IFN-β [15]. Our in vitro data now provide further evidence that IFN-β-mediated induction of GM-CSF is partially dependent on elevated IL-6 expression. In summary, these results show that IFN-β, with IL-23, induces a highly inflammatory cell population.

### 3.3. IFN-β Induces Key Transcription Factors for the Differentiation of Pathogenic T Cells

**Figure 3:**
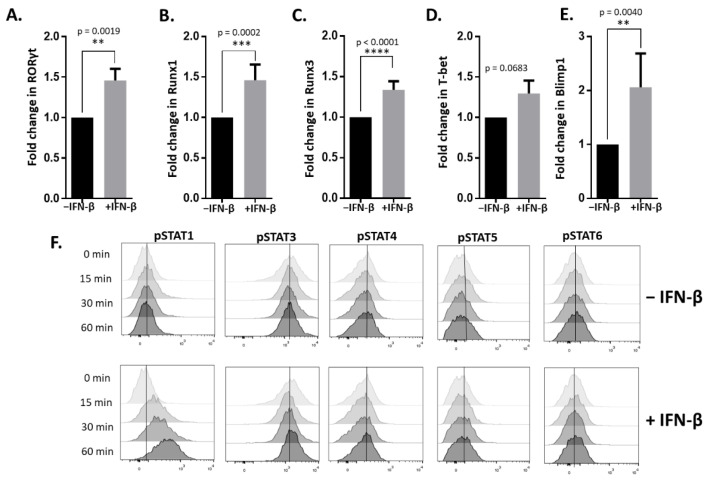
Transcriptional differences in pathogenic IL-23-stimulated TH17 cells induced by IFN-β treatment. Spleen and lymph node cells from MOG_35–55_-immunized C57BL/6J mice were cultured with MOG_35–55_, IL-23 and anti-IFN-γ in the presence or absence of IFN-β for three days. Gene expression was measured by qRT-PCR for the transcription factors; (**A**) RORγt, (**B**) Runx1, (**C**) Runx3, (**D**) T-bet and (**E**) Blimp-1. Cells from the spleen and lymph nodes of MOG-immunized mice were stimulated with MOG_35–55_, anti-IFN-γ, IL-23 ± IFN-β for 0, 15, 30 and 60 min. CD4^+^ T cells were assessed for phosphorylation of (**F**) STAT1, STAT3, STAT4, STAT5 and STAT6 using flow cytometry. The results are representative of two to four independent experiments. Statistical analysis was determined using Mann−Whitney tests. *p* values < 0.05 were considered significant (**** indicates *p* < 0.0001, *** indicates *p* < 0.001, ** indicates *p* < 0.01).

We observed that IFN-β increased inflammatory cytokine production from T-helper cells. Therefore, we determined if transcription factors important for the differentiation of pathogenic T-cell populations were altered by IFN-β. Transcription factors for TH17 and TH1 differentiation have been shown to promote the inflammatory potential of T-cells [19,20,21]. We found that IFN-β significantly upregulated the TH17-lineage transcription factors RORγt and Runx1 (Figure 3A,B), which was consistent with our observation of an increased IL-17 production by IFN-β. We also observed that the TH1-lineage transcription factor Runx3 (Figure 3C), but not T-bet (Figure 3D), was significantly elevated by IFN-β. Interestingly, it has been suggested that cells that have both TH1 and TH17 phenotypes are highly pathogenic. In a recent study, Blimp-1 was identified as a crucial IL-23-induced transcription factor that helped drive TH17-mediated inflammation through the induction of GM-CSF [22]. We observed that Blimp-1 expression was markedly increased in IFN-β- and IL-23-stimulated cells compared to cells stimulated with only IL-23 (Figure 3E). IFN-β signals through the JAK-STAT pathway, primarily through STAT1. However, IFN-β can also activate other STAT transcription factors, including STAT3 [23]. STAT1 signaling is required for TH1 differentiation, and STAT3 is required for TH17 differentiation [24,25]. We found that IFN-β resulted in the intracellular phosphorylation of STAT1 (Figure 3F). We found no induction of STAT3, STAT4, STAT5 or STAT6 phosphorylation by IFN-β (Figure 3F). Our data demonstrate that IFN-β promotes a phenotype that has both TH1 and TH17 characteristics, a phenotype shown to be highly pathogenic [26].

### 3.4. IFN-β Has Differential Effects on TGF-β-Dependent and IL-23-Dependent TH17 Differentiation

**Figure 4:**
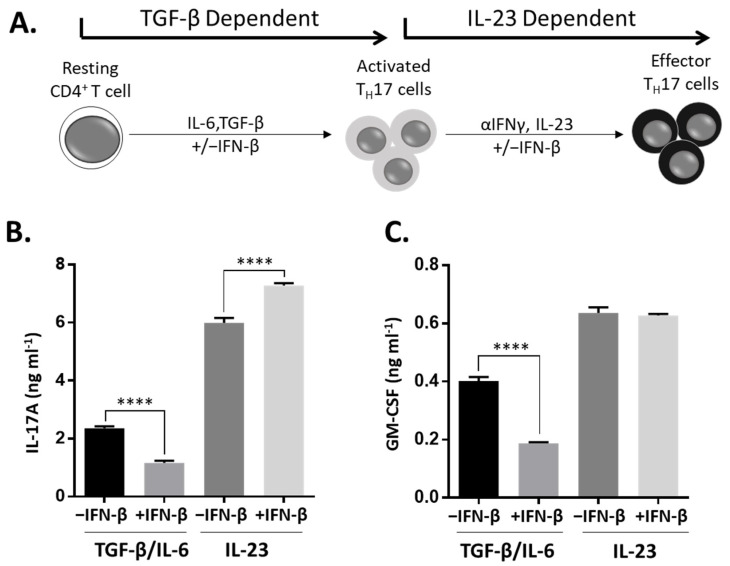
Impact of IFN-β on cytokine production during TGF-β-dependent and IL-23-dependent TH17 differentiation. Spleen and lymph node cells from healthy C57BL/6J were depleted of CD8+ cells and differentiated in the culturing scheme depicted in (**A**). For TGF-β-dependent TH17 differentiation, cells were stimulated with anti-CD3, anti-CD28, IL-6, TGF-β ± IFN-β. For IL-23-dependent TH17 differentiation, cells were stimulated with anti-CD3, anti-CD28, IL-23, anti-IFN-γ ± IFN-β. The concentrations of (**B**) IL-17A and (**C**) GM-CSF secreted were determined by ELISA following early and late differentiation. Statistical analysis was performed using one-way ANOVAs. *p* values < 0.05 were considered significant (**** indicates *p* < 0.0001). Error bars indicate s.e.m.

The data described above contradict reports showing that IFN-β inhibits TH17 development. In culture, TH17 cells can develop in the presence or absence of TGF-β or IL-23 (Figure 4A). TGF-β, with IL-6, initiates the differentiation of naïve T cells to TH17 cells; however, these TH17 cells are not highly inflammatory [27]. IL-23 is also involved with the differentiation of TH17 cells and is essential in driving the inflammatory functions of these cells [27,28]. We speculated that IFN-β may have different effects on these discrete pathways of TH17 differentiation. First, we assessed the effect of IFN-β on TGF-β-mediated TH17 differentiation. We differentiated resting CD4^+^ T cells with IL-6 and TGF-β and found that IFN-β significantly reduced the expression of IL-17 and GM-CSF in these cultures (Figure 4B,C). Next, we assessed the effects of IFN-β on IL-23-mediated TH17 development. Here we took the initial TH17 cultures (TGF-β and IL-6 without IFN-β) and restimulated the cells with IL-23 in the presence or absence of IFN-β. In this condition, we found that IFN-β significantly increased the expression of IL-17 but had no effect on GM-CSF expression (Figure 4B,C). These data demonstrate that TGF-β and IL-23 are important factors that impact the function of IFN-β on TH17 development.

## 4. Discussion

These new data help to rectify the apparent conflicting theories on how IFN-β affects TH17 function. One predominant theory behind the efficacy of IFN-β is that this therapy reduces disease by inhibiting TH17 differentiation and function [4,5,6]. However, MS and NMO patients with high TH17 signatures and mice with TH17-induced EAE have exacerbated disease when treated with IFN-β [2,3,9,11,13,14,29]. Our new data provide key insights into how IFN-β paradoxically increases TH17 pathology. IFN-β has differential effects on discrete pathways of TH17 development. In accordance with previous reports, IFN-β inhibits IL-17 production; however, this is during TGF-β-dependent TH17 development, which does not produce a pathogenic cell population [27]. In contrast, IFN-β enhances the pathological functions of T cells during TH17 development, which is driven by IL-23 [27,28]. Our experiments define a mechanism where IFN-β with IL-23 induces a highly pathogenic T helper cell population that expresses elevated levels of Blimp1, the TH17-lineage genes RORγt and Runx1, and the TH1-lineage gene Runx3. These pathogenic T cells secrete elevated levels of IL-6 which contribute to the secretion of the inflammatory cytokine GM-CSF.

This study provides key insights into how IFN-β drives pathology in diseases with elevated TH17 signatures. Our observations suggest that IFN-β with IL-23 can alter both the transcriptional and cytokine profiles towards an inflammatory phenotype during TH17-mediated disease. These findings provide further evidence that IFN-β may worsen in certain neuro-autoimmune diseases, such as in patients with NMO.

## Data Availability

All data from this manuscript will be provided to anyone upon request.

## Supplementary Materials

The following are available online at https://www.mdpi.com/article/10.3390/cells10082139/s1, Table S1: List of Primers.

## References

[1] Rio J., Nos C., Tintore M., Borras C., Galan I., Comabella M., Montalban X. (2002). Assessment of different treatment failure criteria in a cohort of relapsing-remitting multiple sclerosis patients treated with interferon beta: Implications for clinical trials. Ann. Neurol..

[2] Palace J., Leite M.I., Nairne A., Vincent A. (2010). Interferon Beta treatment in neuromyelitis optica: Increase in relapses and aquaporin 4 antibody titers. Arch. Neurol..

[3] Uzawa A., Mori M., Hayakawa S., Masuda S., Kuwabara S. (2010). Different responses to interferon beta-1b treatment in patients with neuromyelitis optica and multiple sclerosis. Eur. J. Neurol. Off. J. Eur. Fed. Neurol. Soc..

[4] Pennell L.M., Fish E.N. (2014). Immunoregulatory effects of interferon-beta in suppression of Th17 cells. J. Interferon Cytokine Res. Off. J. Int. Soc. Interferon Cytokine Res..

[5] Ramgolam V.S., Sha Y., Jin J., Zhang X., Markovic-Plese S. (2009). IFN-beta inhibits human Th17 cell differentiation. J. Immunol..

[6] Durelli L., Conti L., Clerico M., Boselli D., Contessa G., Ripellino P., Ferrero B., Eid P., Novelli F. (2009). T-helper 17 cells expand in multiple sclerosis and are inhibited by interferon-beta. Ann. Neurol..

[7] Steinberg S.C., Faris R.J., Chang C.F., Chan A., Tankersley M.A. (2010). Impact of adherence to interferons in the treatment of multiple sclerosis: A non-experimental, retrospective, cohort study. Clin. Drug Investig..

[8] Dunn N., Fogdell-Hahn A., Hillert J., Spelman T. (2020). Long-Term Consequences of High Titer Neutralizing Antibodies to Interferon-beta in Multiple Sclerosis. Front. Immunol..

[9] Hegen H., Adrianto I., Lessard C.J., Millonig A., Bertolotto A., Comabella M., Giovannoni G., Guger M., Hoelzl M., Khalil M. (2016). Cytokine profiles show heterogeneity of interferon-beta response in multiple sclerosis patients. Neurol. Nneuroimmunol. Neuroinflamm..

[10] Lee L.F., Axtell R., Tu G.H., Logronio K., Dilley J., Yu J., Rickert M., Han B., Evering W., Walker M.G. (2011). IL-7 promotes T(H)1 development and serum IL-7 predicts clinical response to interferon-beta in multiple sclerosis. Sci. Transl. Med..

[11] Axtell R.C., de Jong B.A., Boniface K., van der Voort L.F., Bhat R., de Sarno P., Naves R., Han M., Zhong F., Castellanos J.G. (2010). T helper type 1 and 17 cells determine efficacy of interferon-beta in multiple sclerosis and experimental encephalomyelitis. Nat. Med..

[12] Hartung H.P., Steinman L., Goodin D.S., Comi G., Cook S., Filippi M., O’Connor P., Jeffery D.R., Kappos L., Axtell R. (2013). Interleukin 17F level and interferon beta response in patients with multiple sclerosis. JAMA Neurol..

[13] Matsushita T., Tateishi T., Isobe N., Yonekawa T., Yamasaki R., Matsuse D., Murai H., Kira J. (2013). Characteristic cerebrospinal fluid cytokine/chemokine profiles in neuromyelitis optica, relapsing remitting or primary progressive multiple sclerosis. PLoS ONE.

[14] Shimizu J., Hatanaka Y., Hasegawa M., Iwata A., Sugimoto I., Date H., Goto J., Shimizu T., Takatsu M., Sakurai Y. (2010). IFNbeta-1b may severely exacerbate Japanese optic-spinal MS in neuromyelitis optica spectrum. Neurology.

[15] Agasing A.M., Wu Q., Khatri B., Borisow N., Ruprecht K., Brandt A.U., Gawde S., Kumar G., Quinn J.L., Ko R.M. (2020). Transcriptomics and proteomics reveal a cooperation between interferon and T-helper 17 cells in neuromyelitis optica. Nat. Commun..

[16] Livak K.J., Schmittgen T.D. (2001). Analysis of relative gene expression data using real-time quantitative PCR and the 2(-Delta Delta C(T)) Method. Methods.

[17] El-Behi M., Ciric B., Dai H., Yan Y., Cullimore M., Safavi F., Zhang G.X., Dittel B.N., Rostami A. (2011). The encephalitogenicity of T(H)17 cells is dependent on IL-1- and IL-23-induced production of the cytokine GM-CSF. Nat. Immunol..

[18] Veldhoen M., Hocking R.J., Atkins C.J., Locksley R.M., Stockinger B. (2006). TGFbeta in the context of an inflammatory cytokine milieu supports de novo differentiation of IL-17-producing T cells. Immunity.

[19] Ivanov I.I., McKenzie B.S., Zhou L., Tadokoro C.E., Lepelley A., Lafaille J.J., Cua D.J., Littman D.R. (2006). The orphan nuclear receptor RORgammat directs the differentiation program of proinflammatory IL-17+ T helper cells. Cell.

[20] Szabo S.J., Kim S.T., Costa G.L., Zhang X., Fathman C.G., Glimcher L.H. (2000). A novel transcription factor, T-bet, directs Th1 lineage commitment. Cell.

[21] Wang Y., Godec J., Ben-Aissa K., Cui K., Zhao K., Pucsek A.B., Lee Y.K., Weaver C.T., Yagi R., Lazarevic V. (2014). The transcription factors T-bet and Runx are required for the ontogeny of pathogenic interferon-gamma-producing T helper 17 cells. Immunity.

[22] Jain R., Chen Y., Kanno Y., Joyce-Shaikh B., Vahedi G., Hirahara K., Blumenschein W.M., Sukumar S., Haines C.J., Sadekova S. (2016). Interleukin-23-Induced Transcription Factor Blimp-1 Promotes Pathogenicity of T Helper 17 Cells. Immunity.

[23] Tanabe Y., Nishibori T., Su L., Arduini R.M., Baker D.P., David M. (2005). Cutting edge: Role of STAT1, STAT3, and STAT5 in IFN-alpha beta responses in T lymphocytes. J. Immunol..

[24] Afkarian M., Sedy J.R., Yang J., Jacobson N.G., Cereb N., Yang S.Y., Murphy T.L., Murphy K.M. (2002). T-bet is a STAT1-induced regulator of IL-12R expression in naive CD4+ T cells. Nat. Immunol..

[25] Yang X.O., Panopoulos A.D., Nurieva R., Chang S.H., Wang D., Watowich S.S., Dong C. (2007). STAT3 regulates cytokine-mediated generation of inflammatory helper T cells. J. Biol. Chem..

[26] Duhen R., Glatigny S., Arbelaez C.A., Blair T.C., Oukka M., Bettelli E. (2013). Cutting edge: The pathogenicity of IFN-gamma-producing Th17 cells is independent of T-bet. J. Immunol..

[27] McGeachy M.J., Bak-Jensen K.S., Chen Y., Tato C.M., Blumenschein W., McClanahan T., Cua D.J. (2007). TGF-beta and IL-6 drive the production of IL-17 and IL-10 by T cells and restrain T(H)-17 cell-mediated pathology. Nat. Immunol..

[28] Langrish C.L., Chen Y., Blumenschein W.M., Mattson J., Basham B., Sedgwick J.D., McClanahan T., Kastelein R.A., Cua D.J. (2005). IL-23 drives a pathogenic T cell population that induces autoimmune inflammation. J. Exp. Med..

[29] Varrin-Doyer M., Spencer C.M., Schulze-Topphoff U., Nelson P.A., Stroud R.M., Cree B.A., Zamvil S.S. (2012). Aquaporin 4-specific T cells in neuromyelitis optica exhibit a Th17 bias and recognize Clostridium ABC transporter. Ann. Neurol..

