# Supplementary material for: Interferon-β Intensifies Interleukin-23-Driven Pathogenicity of T Helper Cells in Neuroinflammatory Disease
Source: Cells. 2021 Aug 20;10(8):2139. doi: 10.3390/cells10082139 (PMC8392231; doi:10.3390/cells10082139)
Supplement: Supplementary file 1 [file cells-10-02139-s001.zip › cells-1314447-SI.pdf]

Supplementary Material

Table S1. List of primers.

Gene	Primer	Primer sequence (5'→3')
T-bet	Forward	TTC AAC CAG CAC CAG ACA G
	Reverse	AGA CCA CAT CCA CAA ACA TCC
Runx3	Forward	AGG TTC AAC GAC CTT CGA TT
	Reverse	GTC CAT CCA CAG TGA CCT TG
RORγt	Forward	TTT CTG AGG ATG AGA TTG CCC
	Reverse	TTG TCG ATG AGT CTT GCA GAG
Runx1	Forward	ACA AGT TGC CAC CTA CCA TAG
	Reverse	CAG AGG AAG AGG TGA TGG ATC
Blimp1	Forward	ATT AAG CCT ATC CCT GCC AAC
	Reverse	CTA CTG TAT TGC TTT GGG TTG C
